# Focus on the Mechanisms and Functions of Pyroptosis, Inflammasomes, and Inflammatory Caspases in Infectious Diseases

**DOI:** 10.1155/2022/2501279

**Published:** 2022-01-29

**Authors:** Haichao Song, Bintong Yang, Ying Li, Aidong Qian, Yuanhuan Kang, Xiaofeng Shan

**Affiliations:** ^1^College of Veterinary Medicine, College of Animal Science and Technology, Jilin Provincial Engineering Research Center of Animal Probiotics, Key Laboratory of Animal Production and Product Quality Safety of Ministry of Education, Jilin Agricultural University, Changchun, Jilin 130118, China; ^2^Marine College, Shandong University, Weihai, Shandong 264209, China

## Abstract

Eukaryotic cells can initiate several distinct self-destruction mechanisms to display essential roles for the homeostasis maintenance, development, and survival of an organism. Pyroptosis, a key response mode in innate immunity, also referred to as caspase-1-dependent proinflammatory programmed necrotic cell death activated by human caspase-1/4/5, or mouse caspase-1/11, plays indispensable roles in response to cytoplasmic insults and immune defense against infectious diseases. These inflammatory caspases are employed by the host to eliminate pathogen infections such as bacteria, viruses, protozoans, and fungi. Gasdermin D requires to be cleaved and activated by these inflammatory caspases to trigger the pyroptosis process. Physiological rupture of cells results in the release of proinflammatory cytokines, the alarmins IL-1*β* and IL-18, symbolizing the inflammatory potential of pyroptosis. Moreover, long noncoding RNAs play direct or indirect roles in the upstream of the pyroptosis trigger pathway. Here, we review in detail recently acquired insights into the central roles of inflammatory caspases, inflammasomes, and pyroptosis, as well as the crosstalk between pyroptosis and long noncoding RNAs in mediating infection immunity and pathogen clearance.

## 1. Introduction

Cells die when they are subjected to endogenous and exogenous noxious stimuli. Cell death triggered by these stimuli includes programmed death and necrosis. Apoptosis, probably well-characterized and most widely recognized programmed cell death, is a normal noninflammatory cell death that depends on particular cysteine-dependent and aspartate-specific proteases, or caspases, and is of great significance for cell growth and differentiation and even the development of the body [1]. Apoptosis mainly relies on apoptotic caspases to initiate and cleave cellular substrates and manifests as cell shrinkage, nuclear chromatin and cytoplasm condensation, DNA lysis, and retention of intact plasma membrane structures. Fragmented apoptotic bodies are engulfed by the surrounding phagocytes. However, with the passage of time, scientists have learned of multiple modes of cell death in addition to apoptosis, such as oncosis, autophagy, ferroptosis, necrosis, and pyroptosis.

Pyroptosis, a recently discovered novel type of inflammatory cell death, is mediated by caspase (also referred to as interleukin-1 beta-converting enzyme) and is distinguished from apoptosis. In 1992, the *Sansonetti* team [2] discovered that *Shigella* can cause death after infecting macrophages and electron microscopy observation results showed that this kind of cell death is characterized by chromatin solidification, cell membrane blebbing, cytoplasmic vacuolation, endoplasmic reticulum expansion, retention of intact organelle structure, and DNA fragmentation in genomic electrophoresis, which was incorrectly classified as “apoptosis.” In 1994, the team found that the “apoptosis” of the macrophages in *Shigella* infection was accompanied by a large amount of IL-1 released without IL-6 or TNF-*α* and it was believed that this kind of “apoptosis” was a manner of death accompanied by inflammation [3]. In 1996, Chen et al. [4] found that caspase-1 was activated in the death of macrophages and inhibition of its activation prevented cell death. In the following decades, other researchers continued to help characterize the discovery of this “apoptosis,” which was the term attributed to this inflammatory cell death. In 2000, *Brennan* and *Cookson* [5] found that the cell death of macrophages after *Salmonella typhimurium* infection was significantly different from traditional apoptosis. Subsequently, *Boise* and *Collins* [6] summarized the discoveries that they made in the past ten years and concluded that cell death caused by caspase-1 is a completely different mode of death than traditional apoptosis—the cell death that they had been studying was faster, the cell membrane integrity was destroyed, and the inflammatory process was obvious [4, 5, 7, 8]. Some processes, however, were indistinguishable from apoptosis. Later, the term pyroptosis (from combining Greek “pyro” for fire or fever and “ptosis” for falling) was used to define this mode of cell death [9].

Pyroptosis is a proinflammatory and lytic cell death mediated by inflammatory caspase-1/4/5/11 [10], and it is mainly manifested by plasma membrane rupture which causes K^+^ to flow out and water molecules to infiltrate [11, 12] and the release of proinflammatory intracellular contents. In this process, from the reception and conduction of dangerous signals to the initiation of pyroptosis, the transmission of signals from extramembrane receptors to intracytoplasmic inflammasomes and related signal activation are inseparable. GSDM family proteins play important roles as downstream signals of pyroptosis triggers. However, in recent years, an increasing number of studies have shown that, in addition to the classic research results showing that GSDMD is cleaved to serve as an executioner protein, there are many alternative pyroptosis triggers. Similarly, the inflammasome, as the upstream sensing signalling hub, is associated with various types of signals associated with unique mechanisms. Therefore, this review mainly discusses the assembly, signalling, and regulation of proteins involved in the process of pyroptosis and introduces some new pathways discovered in recent years that trigger pyroptosis, in addition to the known findings, as well as the roles of lncRNAs in the process of pyroptosis. We hope to provide some reference and evidence to support future research on various aspects of pyroptosis.

## 2. Canonical and Noncanonical Pathways of Pyroptosis

**Figure 1:**
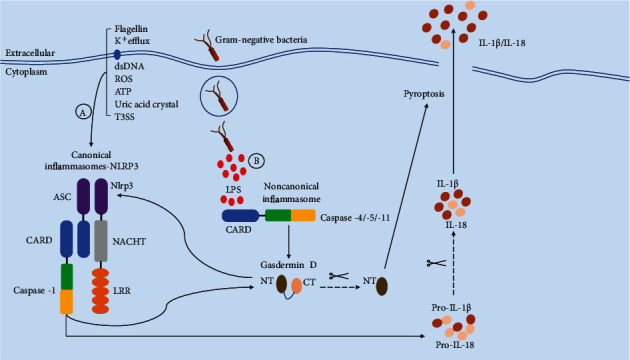
Canonical and noncanonical activation modes of pyroptosis. (A) Canonical pyroptosis activation, also known as caspase-1-dependent pyroptosis involving pathogen-associated molecular patterns (PAMPs) or host-derived danger signals (danger-associated molecular patterns (DAMPs)), is recognized by the corresponding intracellular NOD-like receptors (NLRs), and a large number of adapter ASCs and the cysteine protease caspase-1 are recruited to form their respective inflammasomes and macromolecular complexes. During this process, an abundance of recruited procaspase-1 cleaves GSDMD to generate N-terminal and C-terminal GSDMD domains, disrupting the autoinhibitory equilibrium state of GSDMD and the N-terminal domain binds to the cell membrane to form an inner plasma membrane pore with a size of 12–14 nm. The formation of pores disrupts the homeostasis of the environment inside and outside the cell. External sodium ions enter the cell, resulting in an imbalance of osmotic pressure, causing a large influx of water and swelling of the cell, which eventually leads to cell lysis. Simultaneously, the pores are large enough to allow mature IL-1*β* (4.5 nm) and IL-18 (5.0 nm) to be secreted from the internal environment at a slow rate, causing local immune responses and amplifying inflammatory responses. (B) In noncanonical pyroptosis, also referred to as caspase-1-independent pyroptosis, caspase-11 in mouse cells and caspase-4/5 in human cells directly induce the activation of cytoplasmic LPS in infected cells through direct and specific binding between lipid A in LPS and the CARD in the caspase. Similar to the canonical pyroptosis mode, a large number of recruited procaspase-4/5/11 proteins are activated by oligomerization and directly cleave GSDMD to initiate pyroptosis; however, this process does not involve the release of proinflammatory cytokines. In addition, the GSDMD N-terminal domain generated in this process can activate the NLRP3 inflammasome, triggering caspase-1-dependent canonical pyroptosis.

Canonical pyroptosis is a form of necrotic cell death mediated by activated caspase-1 [13]. The initiation of pyroptosis can be traced back to danger-associated molecular patterns (DAMPs) (such molecules including bacterial flagella, lipopolysaccharide (LPS) of Gram-negative bacteria, and bacterial toxins) and pathogen-associated molecular patterns (PAMPs) (such molecules include uric acid crystals and cytoplasm) [14]. Some bacterial toxins and dangerous small-molecule substances can be sensed by extracellular receptors and transmitted to the cell to trigger the assembly of the corresponding inflammasome, the specific supramolecular complex, including NLRP1, NLRP3, NLRC4, AIM2, and pyrin. The interaction between PRR and caspase-1 is mediated by the adaptor protein ASC through its pyrin domain (PYD) that interacts with PRR and its CARD that interacts with caspase-1. Activated PRRs, such as AIM2 and NLRP3, induce the formation of PYD filaments with ASC, which clusters the CARD of ASC to nucleate the CARD filaments of caspase-1 to form structures called “specks” and inducing the activation and cleavage of caspase-1 [15]. Intracellular pathogens enter the cytoplasm through a specialized evolutionarily conserved mechanism (vesicular endocytosis or receptor-binding endocytosis) [16] and are recognized by NOD- (nucleotide-binding oligomerization domain-) like receptors, triggering similar responses in the assembly of intracellular inflammasomes [17, 18]. Activated caspase-1 cleaves GSDMD of the GSDM family protein to trigger cell lytic death, while converting pro-IL-18 and pro-IL-1*β* into their mature forms IL-18 (5.0 nm) and IL-1*β* (4.5 nm), respectively [12, 19] (Figure 1(a)).

**Figure 2:**
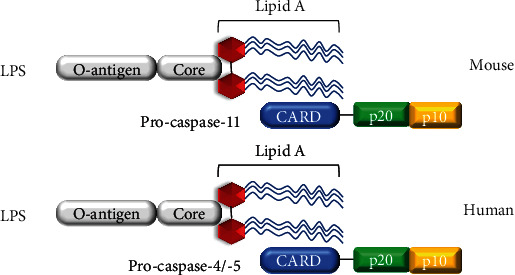
Caspase-4/5 and 11 is activated by directly sensing LPS. The CARD domain of mouse caspase-11 or human caspase-4/5 can directly recognize lipid A of lipopolysaccharide (LPS). After sensing intracellular LPS, caspase-4/-5 and 11 are recruited in large numbers and activated by oligomerization.

The noncanonical pyroptosis pathway is initiated after intracellular receptors sense cytoplasmic LPS [20], which depends on the formation of the macromolecular signal complexes composed of caspase-4/5 or 11 in the cytoplasm called “noncanonical inflammasomes” [21, 22]. Correspondingly, activated caspases can directly cleave GSDMD, initiating pyroptosis, and the newly cleaved GSDMD N-terminal fragment can activate the NLRP3 inflammasome of the canonical pathway and trigger pyroptosis (Figure 2). These two pathways share similarities and exhibit differences, coordinating with each other in the innate immune response to play roles. Moreover, the expression of murine caspase-11 is highly inducible by multiple proinflammatory stimuli such as TLR ligands, poly(I:C), IFNs, and Pam3CSK4, while human caspase-4/5 are constitutively expressed in macrophages, monocytes, and various additional cell types [23–25]. Notably, Gram-negative bacteria in the cytoplasm are more likely to trigger the abovementioned noncanonical pathway of pyroptosis, mainly because of the activation of acetylated lipid A (a component of Gram-negative bacteria LPS) induced by special intracellular caspases, but it is not found in Gram-positive bacteria (Figure 1(b)).

## 3. Receptor Activation and Signal Transduction

The host relies on the immune system to resist invading pathogenic microorganisms. In vertebrates, innate immunity and adaptive immunity work together. Although both immune pathways recognize “non-self” molecular patterns through specific receptors, their triggering mechanisms are different. Innate immunity, as the host's first line of defense against foreign pathogens, can be quickly activated after infection, relying on immune cells such as macrophages and leukocytes to engulf and destroy foreign microorganisms and harmful substances in a pathogen-specific recognition manner. In the late 1980s, Jane called the pathogen-specific and evolutionarily conserved key molecular patterns recognized by the innate immune system as PAMPs [26, 27] and they include structural components of bacteria, such as LPS, peptidoglycan (PGN), and viral nucleic acid molecules (DNAs or RNAs). In addition, it was predicted that the host expresses PAMP receptors, called pattern recognition receptors (PRRs) [26, 28]. PRR recognition of PAMPs can stimulate a series of defense mechanisms in the host, such as complement activation, phagocytosis, and the expression of proinflammatory molecules. In addition, PRRs can recognize host-derived danger signals or DAMPs. This section reviews the latest information on the detection of pathogenic microorganisms through PRRs and the signalling transduction mechanism of pyroptosis.

### 3.1. Toll-Like Receptor (TLR) Classification and Recognition

TLR is a type I transmembrane protein composed of extracellular leucine-rich repeats (LRRs), which mediate the recognition of PAMPs, transmembrane domains, and a cytoplasmic Toll interleukin receptor (TIR) domain that interacts with downstream adaptor proteins required for signal transmission. To date, ten and twelve TLR family members have been identified in humans and mice, respectively (Table 1). Among these proteins, TLR1–TLR9 are conserved in mice and humans and TLR10 in mice is nonfunctional. TLR11, TLR12, and TLR13 do not exist in the human genome [29–31]. TLRs are roughly classified into two categories according to their cell location and the PAMPs they recognize. TLR1, TLR2, TLR4, TLR5, TLR6, and TLR10 are located on the plasma membrane and recognize lipids, lipoproteins, and proteins, while TLR3, TLR7, TLR8, TLR9, TLR11, TLR12, and TLR13 are located in the endosomal compartment where they recognize microbial nucleic acids and their own nucleic acids under certain conditions (autoimmunity) [32, 33].

As a TLR on the cell surface, TLR4 has been identified as a receptor for bacterial LPS (a component of the cytoderm of Gram-negative bacteria and can cause septic shock) [34]. TLR4 can bind to myeloid differentiation factor 2 (MD2) on the cell surface to recognize LPS, and crystal structure studies indicate that the two copies of TLR4-MD2-LPS interact symmetrically to form a TLR4 homodimer [35]. After ligand binding, TLR4 is transported into the cell through a dynein-dependent mechanism, thereby inducing a TRIF-dependent pathway [36, 37]. As another surface receptor, TLR2 specifically recognizes a variety of microbial components derived from bacteria, fungi, viruses, and mycoplasma and can detect lipoproteins, peptidoglycans, lipoprotein acids, zymosan, and mannose in Gram-positive bacteria [29, 38, 39]. TLR2 mediates the recognition of PAMPs and subsequent signal transduction through heterodimerization with TLR1 or TLR6 on the plasma membrane. TLR2/TLR1 and TLR2/TLR6 recognize triacylated lipoproteins and diacylated lipoproteins, respectively, and induce the production of various proinflammatory cytokines except type I interferons (IFNs). TLR5 recognizes bacterial flagellin from virtually all motile bacteria [40, 41] and can also recognize an unknown protein component of uropathogenic *Escherichia coli* (UPEC) [42].

### 3.2. TLR Signalling

As a key innate immune receptor, Toll-like receptor (TLR) family proteins are among the first pattern recognition receptors to be studied and play central roles in mediating the host's antipathogen infection process [43]. Mammalian TLRs, as transmembrane proteins, contain multiple leucine-rich repeats (LRRs) in the extracellular region, which are critical for recognizing PAMPs. The intracellular part mainly regulates the TIR (Toll/IL-1 receptor) domain of protein-protein interactions to mediate signal transduction, which is mainly initiated by the organization and recruitment of four different TIR domain-containing structural adaptor proteins: myeloid differentiation factor 88 (MyD88), MyD88 adaptor-like protein (MAL/TIRAP), IFN-*β* (TRIF, or TICAM-1), and TRIF-related adaptor molecules (TRAM) [44]. The signal transduction of TLRs depends on two pathways, MyD88 and TRIF, which depend on the adaptor protein that is activated. In addition to TLR3, all TLRs rely on MyD88 to activate NF-*κ*B and MAPK signalling pathways to induce the expression of proinflammatory genes and the secretion of related cytokines [45, 46]. TLR2, TLR3, and TLR4 rely on the TRIF pathway to activate the IRF3 and NF-*κ*B pathways and induce the production of type I interferons and other inflammatory cytokines [26, 47, 48].

### 3.3. Intracellular Signalling and Pyroptosis

Another type of PRR comprises intracellular receptors, including RIG-1-like receptors (RLRs), AIM2-like receptors, and NLRs [44] (Table 2). Nod-like receptors (NLRs) play key roles in recognizing danger signals in the cytosol. Proteins containing NLR nucleotide-binding oligomerization domains (NOD1 and NOD2) can induce signalling cascades similar to those initiated by TLRs after ligand recognition [49, 50], leading to the secretion of inflammatory cytokines [16], such as tumor necrosis factor (TNF), IL-6, IL-8, and type I interferon (IFN). On the other hand, other NLRs trigger the activation of cysteine protease, caspase-1, inducing pyroptosis and the secretion of inflammatory cytokines IL-1*β* and IL-18 [51, 52]. Interestingly, TLRs and NLRs that activate caspase-1 often work together and both can stimulate the production of pro-IL-1*β* and promote its accumulation in cells [53–55]. Eventually, caspase-1 is activated and a large amount of IL-1*β* is secreted in response to danger signalling derived from cytoplasmic pathogens. Interestingly, NOD1 can respond to LPS stimulation similar to the response of plant R protein receptors to pathogenic microbial components, which play key signal transduction roles in immune signalling pathways [56]. Further research revealed that NOD1 can be activated by the PGN fragment DAP (*γ*-D-glutamyl-meso-diaminopimelic acid) which exists on the cytoplasm of Gram-negative bacteria and certain Gram-positive bacteria, while NOD2 recognizes the Gram-negative PGN fragment MDP (muramyl dipeptide) which exists in both Gram-negative bacteria and Gram-positive bacteria [57–60].

## 4. Inflammasomes

### 4.1. Classification and Structure of the Inflammasome

**Figure 3:**
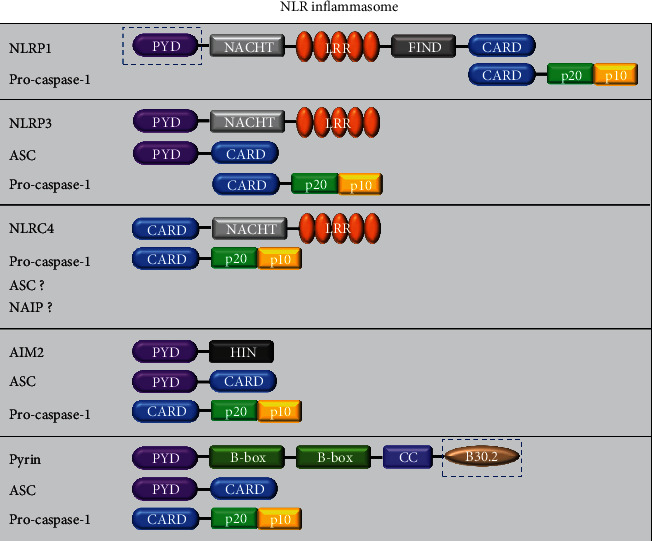
NLRP1, NLRP3, NLRC4, AIM2, and pyrin inflammasomes. NLRP1 was the first inflammasome complex formed by NLRs to be discovered. The findings were mainly in humans and mice. In humans, there is only one NLRP1 protein consisting of an amino-terminal PYD, an NAPDH, LRRs, a functional domain (FIND), and a carboxy-terminal domain (CARD). In rodents, such as mice, there are multiple orthologues of NLRP1 but they lack the PYD, as indicated in the black dashed box. NLRP3, also known as cryopyrin, is the best characterized inflammasome and is mainly composed of NLRP3 receptor protein (consisting of the three domains of PYD, NACHT, and LRR), ASC (PTD and CARD domains), and procaspase-1 protein (consisting of the CARD domain and caspase-1). NLRC4 mainly recognizes bacterial flagella and the type III secretion system (T3SS). It needs stimuli only by the NLRC4 receptor protein and procaspase-1 to be activated. ASC is optional, but maximum ASC activation is required. Upon *Legionella pneumophila* infection, NAIP is involved in the activation of NLRC4. The AIM2 inflammasome harbours an N-terminal PYD and a C-terminal HIN. Pyrin has a PYD, two B-boxes, and a coiled-coil domain. Compared with mouse pyrin, human pyrin has an addition C-terminal B30.2 domain, which is also known as the SPRY/PRY domain.

Inflammasomes are multiprotein complexes in the cytoplasm that are assembled by the PRRs [61]. To date, five receptor proteins have been confirmed to assemble into inflammasomes, including the nucleotide-binding oligomerization domain (NOD), leucine-rich repeat (LRR) sequence protein members (NLRP1 [62], NLRP3 [63], NLRC4 [64, 65]), AIM2 (melanoma deficiency factor) [66], and pyrin [67, 68] (Figure 3). These inflammasomes can be assembled in canonical pathways, and they can also be supplemented by noncanonical pathways mediated by targeting mouse caspase-11 or human caspase-4/5. In addition, poorly functional NLRP6 [69, 70], NLRP7 [71], NLRP12 [72], retinol inducible gene I (RIG-1) [73], and interferon-*γ*- (IFN-*γ*-) inducible proteins [74] can promote the activation of caspase-1. With the in-depth research, inflammasomes have gradually been found to play key roles in regulating multiple cell death modes, such as pyroptosis and apoptosis.

Inflammasomes are assembled after the corresponding signal recognition described above, especially when the intracellular recognition receptor recognizes a pathogen PAMP or DAMP in the cytoplasm. As generally accepted intracellular recognition receptors, members of the NLR family play an indispensable role in the assembly of inflammasomes. The NLR family is mainly composed of a carboxyl terminally bound leucine-rich repetitive sequence (LRR), nucleotide-binding oligomerization domain, and amino-terminal cysteine recruitment binding domain (CARD) [75]. The ALR family contains PYD and at least two HIN-200 domains [76]. Once these two intracellular recognition receptors are activated, they oligomerize at their respective CARD and HIN-200 domain. In turn, the interaction between the PYDNLR domain of the apoptosis-related speck-like protein (ASC) and the PYD of an ALR family protein and the ASC protein induces the extensive recruitment and aggregation of the adaptor protein ASC [77]. In addition, the procaspase-1 recruited by ASCs through the CARD also accumulates in abundance and then synthesizes multimeric inflammasome complexes in the cell. Certain inflammasomes (such as NLRP1 and NLRC4) can induce the assembly of inflammasomes without ASC, using a CARD domain to directly recruit procaspase-1 [78, 79]. However, the participation of ASCs can significantly improve the production efficiency of IL-1*β* and IL-18.

### 4.2. The Relationship between the Inflammasome and Pyroptosis

To date, five types of inflammasome protein complexes have been discovered: NLRP3, NLRP1, NLRC4, AIM2, and pyrin, which induce pyroptosis in different ways. Although the functions and mechanisms of these types of inflammasomes are well understood, the trigger mechanisms are discussed in more detail below.

### 4.3. The NLRP1 Inflammasome

As the initially discovered inflammasome complex formed by the assembly of NLR, NLRP1 is encoded only in humans; however, there are paralogues in rodents, such as NLRP1A-C in mice (lacking the PYD domain) [80]. Two of the five NLRP1b alleles in mice are involved in the recognition of *Bacillus anthracis* lethal toxin (which is composed of four parts: a protective antigen (PA), cell-binding protein, oedema factor, and lethal factor (LF) [80, 81]. The lethal factor is a nonmetallic protease with a zinc-containing metalloprotease-like consensus sequence, which plays a key role in the activation of NLRP1b. The lethal factor is transferred to the cytoplasm through the pore formed by the protective antigen protein. On the one hand, mitogen-activated protein kinase (MAPK) is cleaved to silence the immune signal, and on the other hand, NLRP1b is cleaved. Interestingly, NLRP1b is selective in response to lethal factors, indicating that its activation undergoes an additional process [82, 83]. There may be such an automatic processing event in FIIND, which is essential for the activation of NLRP1b, and only the reactive allele of the lethal factor will undergo automatic proteolysis [84].

### 4.4. The NLRP3 Inflammasome

As the most widely studied inflammasome, NLRP3 responds to a variety of different stimuli, including crystals and particulate matter (such as uric acid crystals, silica, asbestos and alum), extracellular ATP, RNA-DNA complexes, pore-forming toxins, and most bacterial, viral, fungal, and protozoan pathogens [85–87]. Canonical NLRP3 activation mainly includes the following reactions: (1) the outflow of intracellular potassium ions [88], (2) the production of mitochondrial reactive oxygen species (ROS) [89], (3) cathepsin release caused by lysosomal imbalance [90], (4) changes in intracellular levels of the first three [91], and (5) NLRP3 relying on the internal migration of the adaptor protein ASC within the mitochondria. However, some new activation mechanisms have been discovered after years of research, such as the activation of Fas death domain-related protein and caspase-8 in fungal and Gram-negative bacterial infections [92, 93] and CD36-mediated activation of NLRP3 [94]. The deubiquitination of NLRP3 also has a certain effect on its own activation [95]. In all these NLRPs activation mechanisms, the release of potassium ions plays a key role. Activation of NLRP3 requires only a low amount of potassium ions; however, whether NLRP3 directly responds to low concentrations of sodium ions in the cell and whether this response is regulated by other pathways remain to be determined by further studies.

### 4.5. The NLRC4 Inflammasome

NLRC4, which contains a CARD motif similar to NLRP1b, was initially identified due to its similarity to apoptotic protease activator 1, but its induction of pyroptosis does not necessarily depend on ASC [96]. In the host, NLRC4 plays a key role as one of the main inflammasomes against facultative intracellular bacteria [97]. Currently, there are two main mechanisms for its activation: (1) NAIP (inhibitor of apoptosis protein), a homologous ligand of the NLR family, has a BIR motif in the N-terminus, which play important roles in identifying the structure of the corresponding ligand in bacteria. Among them, NAIP2 binds to the rod protein T3SS [98–100], NAIP1 binds to the needle tube proteins of the T3SS, and NAIP5 and NAIP6 bind to bacterial flagellin and then bind to NLRC4 to promote the activation of caspase-1 and (2) Ser533 in NLRC4 is phosphorylated and activated after *Salmonella* infection [101, 102]. Although these two activation mechanisms have been revealed based on the existing evidence, further studies are needed to supplement the description with details.

### 4.6. The AIM2 Inflammasome

AIM2 is a typical inflammasome assembled by the intracellular receptor ALR [103]. When intracellular pathogen DNA is detected by the HIN200 domain of AIM2, such as the DNA of *Francisella tularensis* and vaccinia virus [75, 104], AIM2 will recruit ASCs to activate caspase-1. The AIM2 receptor is a member of the PYHIN (containing pyrin and HIN domains) family [105, 106] and is characterized by its tight binding to oligonucleotides or the oligosaccharide folds between an N-terminal PYD and a C-terminal HIN domain. Studies have shown that the absence of AIM2 or casapse-1 in mice leads to the inability to eliminate *F. tularensis*, which fully reflects the important role of AIM2 and caspase-1 in the host defense of pathogenic microorganisms [107, 108]. Other studies have shown that AIM2 can bind to NLRP3 and NLRC4 to facilitate the activation of caspase-1 in *Listeria monocytogenes* infection [109]. In addition, AIM2 inflammasomes are closely related to human diseases, such as psoriasis and systemic lupus erythematosus, which are all related to the increased expression of AIM2 [110]. Moreover, mice with AIM2 knockout are highly sensitive to the occurrence and development of colon cancer.

### 4.7. The Pyrin Inflammasome

Pyrin, also known as marenostrin or TRIM20, is encoded by the MEFV gene, which is a most recently discovered member of the inflammasome family and has a PYD, two B-boxes, and a coiled-coil domain [111]. There are significant differences in the structure of human and mouse pyrin, that is, human pyrin contains the C-terminal B30.2 domain (also known as the SPRY/PRY domain), and the mutation of this domain is related to autoinflammatory familial Mediterranean fever (FMF) [112]. Chae et al. [113] experimentally proved that Mefv^−/−^ macrophages can normally activate inflammasomes in response to activators, while FMF is caused by acquired functional mutations in Mefv. Pyrin plays an important physiological role in the assembly triggered by bacterial toxins (such as *Clostridium difficile* toxin B and *Clostridium botulinum* toxin C3) and effector proteins (VopS of *Vibrio parahaemolyticus* and lbpA of *Haemophilus*) [68, 114]. However, to date, the activation of AIM2 caused by bacterial infection still needs further study and exploration because the previous findings illustrate only the biological relevance of AIM2 in host defense.

### 4.8. Noncanonical Inflammasomes

In contrast to that of canonical inflammasomes, the activation of noncanonical inflammasomes relies primarily on intracellular LPS [22, 115]. Intriguingly, caspase-4/5 and 11 initiate pyroptosis similarly to caspase-1 upon activation, but they do not cleave pro-IL-1*β* or pro-IL-18. The secretion of mature cytokines is nevertheless observed, as GSDMD-NT generated by the activation of these caspases triggers the assembly of the NLRP3 inflammasome [116]. Surprisingly, caspase 11-induced pyroptosis has been shown to mediate LPS-induced lethality in a mouse model of septic shock induced by endotoxemia and *E. coli*, which was previously thought to be caused by TLR4 [117]. Therefore, host cells have developed two independent systems to recognize LPS outside and inside the cell.

Similar to the assembly of canonical inflammasomes, LPS recognition and caspase-11 recruitment are mediated by a CARD-containing receptor. Surprisingly, both CARD motif of procaspase-11 in mouse macrophages and procaspase-4/5 in human macrophages can directly bind to the lipid A tail of LPS [118, 119]. However, the structure of the LPS and CARD complex is currently unclear and it will be interesting to determine the structural similarity of LPS proteins from different sources. Moreover, once confirmed, this mechanism will reveal a new activation mode for inflammatory caspases. In addition, Benaoudia and colleagues discovered extra players in the human noncanonical inflammasome using a CRISPR library screen; the only strongly positive hit apart from the known components caspase-4 and gasdermin D was interferon regulatory factor-2 (IRF2) (a transcriptional activator of caspase-4) [120]. Without IRF2, induction of IRF1 could substitute to maintain caspase-4 expression.

## 5. Caspase Family Proteins

### 5.1. Source, Structure, and Classification of Caspase Family Proteins

The term caspase is the abbreviated form of cysteine-containing aspartate protease, which is a cytoplasmic protease that has cleavage activity after activation [121, 122]. Initially, caspase was highly homologous to the amino acid sequence of Ced-3, a key protease involved in regulating apoptosis in *Caenorhabditis elegans* and subsequent protein verification revealed that the protein sequence of caspase has a high degree of homology with mammalian interleukin-1-converting enzyme. Therefore, the caspases were initially named CED-3/ICE family proteins [123]. Subsequently, a series of homologous proteases were identified in mammals. Although the caspase family of proteins contains many members involved in different cellular processes, they have almost the same activation mode. Therefore, caspase-1 is described here as an example to illustrate the composition of the caspase structure.

Caspase-1 is synthesized from a protein precursor of 45 kDa, which has biological cleavage activity when activated in its mature form. X-ray crystallographic analysis of the three-dimensional structure indicated that activated caspase-1 is a tetramer composed of two p20 and two p10 subunits [124]. In addition, the proteases in this family containing caspase-1 have the following characteristics: (1) they always cleave the substrate downstream of an aspartic acid, (2) its enzymatic activity depends on the nucleophilicity of cysteine residues, and (3) the protein usually exists in the form of a zymogen. As people's understanding of this protein family continues to increase, there has been a surge in the number of proteins added to this family, causing confusion with the naming convention. As a cysteine protease with aspartic acid specificity, caspases were uniformly named caspase-1 through 10. This naming convention has undoubtedly caused considerable controversy. Moreover, the choice of “caspase” as the root name of all families is based on the two catalytic properties of the enzyme: “c” reflects its catalytic mechanism and “aspase” refers to its ability to cleave aspartic acid residues, which is the distinctive characteristic of this protease. However, to date, the related functional roles of caspase family proteases have been basically clear; they play key roles in mammalian apoptosis and pyroptosis. Since this review focuses on summarizing the caspase family proteins involved in pyroptosis, the following description mainly introduces the caspases that contribute or mediate pyroptosis.

The first reported member of the caspase family was caspase-1, which acts as a processing enzyme of interleukin-1*β* and 18 to mediate the release of the mature forms of IL-1*β* and IL-18. With the advances in later research, it was discovered that caspase-1, 2, 3, 6, 7, 8, 9, 11, 12, and 14 exist in mice and caspase-1, 2, 3, 4, 5, 6, 7, 8, 9, 10, and 14 are present in humans, which is significantly different from the single caspase activation in *Caenorhabditis elegans* [125]. Based on the function of a caspase, it can be broadly classified as an inflammatory caspase or an apoptotic caspase. Inflammatory caspases include caspase-1, 4, 5, 11, and 12, of which the human genome encodes caspase-1, 4, 5, and 12 and the mouse genome encodes caspase-1, 11, and 12. Human caspase-4/-5 and murine caspase-11 are homologues. Apoptosis, as the earliest discovered and identified mode of programmed cell death, mainly involves the activation of initial caspases (including caspase-2, 8, 9, and 10) and effector caspase (caspase-3, 6, and 7). In recent years, people have classified these caspases into three categories based on sequence homology and P4 residue preference [126]. The first type has the prodomain containing CARD and is biased towards having tryptophan or leucine at the P4 position, such as in human inflammatory caspase-1, 4, and 5. The second category has a shorter prodomain and preferentially favors aspartic acid at the P4 position, such as in human apoptosis-related caspase-3/7 and its homologues, *Caenorhabditis elegans* apoptotic protein CED-3, and human caspase-2. The third type of caspase has a longer prodomain, with leucine or valine being the most frequent residue at P4, such as in the human apoptosis promoters caspase-8, 9, and 10. Furthermore, whether there are caspase family proteins in addition to those listed above remains determined.

### 5.2. Inflammatory Caspase Proteins Involved in Pyroptosis and Its Activation Mechanism

In normal cells, caspase family proteins are usually in the form of inactive procaspase and only after amino hydrolysis can they be converted to their biologically active form (with two large and two small subunits that assemble into a tetramer). To date, fifteen caspase species have been confirmed in mammals, thirteen species in humans and eleven in mice. Follow-up in-depth research revealed that these caspases can be further categorized into apoptotic and inflammatory caspases based on their differences in structure and function. Caspase-3 is the representative caspase related to apoptosis. In addition, caspase-2, 3, 6, 7, 8, and 9 also contribute to the process of apoptosis. Inflammatory caspases include caspase-1, 4, 5, 11, 12, 13, and 14, which mediate inflammation. Among these caspases, the well-known canonical pathway species caspase-1 and the non-canonical pathway species caspase-11 (caspase-4/-5 exist in humans) are involved in pyroptosis.

Inflammatory caspases are activated by inflammasomes to trigger pyroptosis, in the canonical pathway mainly mediated by caspase-1 and in the noncanonical pathway mediated by human caspase-11 or mouse caspase-4/5 [127]. Upstream danger signals, such as certain pathogens, toxins, RNAs, or DNAs, stimulate the oligomerization of intracellular PRRs to form a multiprotein complex inflammasome composed of PRR, apoptosis-related speck protein ASC, and procaspase-1. ASC, composed of CARD (a caspase activation and recruitment domain) and PYD, is formed by the interaction of CARD-CARD and PYD-PYD, which can act as double linker proteins that form a molecular bridge to connect inflammasomes with procaspase-1, cleaving the dimer form of procaspase-1 into p10 and p20 subunits to generate caspase-1 with catalytic activity. Caspase-4/5 or 11, which are composed of P20, P10, and special CARD, can directly bind intracellular LPS. The CARD of caspase-4/5 or 11 can interact with the lipid A part of LPS exposed in the cytoplasm [128, 129]. However, in contrast to caspase-1-mediated pyroptosis, caspase-11 or 4/5 cannot cleave pro-IL-18 or pro-IL-1*β* to generate their mature forms. Furthermore, caspase-1 is subject to the regulation by caspase-11 and 4/5. Caspase-11 expression is lost in two Casp1^−/−^ mouse mutant lines [115]. However, Zanoni et al.[130] found that oxidized phospholipids (oxPAPCs) in dendritic cells can induce caspase-11-dependent IL-1*β* secretion without mediating pyroptosis. Moreover, studies have shown that oxPAPC can act as a competitive inhibitor of LPS to inhibit LPS binding to caspase-11, blocking subsequent pyroptosis [131]. Recent studies have also found that caspase-1 can induce the transition from pyroptosis to apoptosis in GSDMD-deficient cells, and caspase-1-induced apoptosis involves the Bid-caspase-9-caspase-3 axis, which can be followed by GSDME-dependent secondary necrosis/pyroptosis [132].

In addition, related studies have shown that cell death induced by caspase-11 is related to the elimination of intracellular bacterial pathogens [133, 134]. When macrophages are pretreated with LPS or IFN-*γ* and then infected with flagellin-deficient mutant *Legionella pneumophila*, the macrophages rapidly undergo caspase-11-dependent pyroptosis [135]. However, wild-type *L. pneumophila* can induce caspase-11-dependent pyroptosis when it infects macrophages pretreated with LPS in vitro, probably because the bacteria are restricted to being in a stable state with intact structure and do not enter the cytosol [136, 137]. Similar to that on caspase-1, research on the function of caspase-11 also focuses on macrophages.

### 5.3. Casapse-8 Plays a Dual Role

Many studies have shown that caspase-8 plays a dual role in the process of regulating innate immunity [138]. Caspase-8 not only can induce the assembly of inflammasomes [139, 140] and activate inflammatory factors to mediate pyroptosis [141–143] but can also inhibit the activation of inflammasomes [144] and necroptosis to inhibit the body's innate immunity [145]. Recent studies have shown that caspase-8 can also directly cleave IL-1*β* precursors through its enzymatic activity, similar to caspase-1, to promote the secretion of IL-1*β*. In 2008, Jonathan et al. [146] found for the first time that overexpression of TRIF (a common adaptor protein of TLR3 and TLR4) or stimulation of TLR3 and TLR4 with poly (I:C) or LPS can induce caspase-8 to directly cleave and activate IL-1*β* at the ASP117 site. Other studies have shown that SMAC (a caspase activator) analogue treatment or knockout of IAPs (inhibitors of apoptosis) in macrophages can activate RIPK3 and mediate caspase-8- and caspase-1-dependent IL-1*β* activation [147]. Additionally, the combination of FAS and FAS ligand (FasL) is an important pathway to the initiation of the direct activation of IL-1*β* by caspase-8. In this pathway, the adaptor protein FADD (Fas-associated protein with death domain) recruits caspase-8 to bind to Fas and participate in the production of mature IL-1*β* [148, 149]. In addition, caspase-8 can indirectly produce IL-1*β* with biological activity by activating caspase-1 to initiate inflammasome assembly [150, 151]. Moreover, caspase-8 is the initiator caspase of extrinsic apoptosis and inhibits necroptosis mediated by RIPK3 and MLKL [152, 153].

Philip [150] found that caspase-8 can promote the expression of c-Rel-dependent inflammatory factors, upregulate the phosphorylation of I*κ*B kinase (IKK) after LPS treatment of macrophages, and ubiquitinate I*κ*B*ε* subunits, which are subsequently degraded by proteolytic enzymes to relieve the inhibitory effect on the c-Rel homodimer. Subsequently, caspase-8 can promote c-Rel nuclear translocation through enzyme activity and scaffold function, and after entering the nucleus, c-Rel is recruited to the caspase-8-dependent promoter to initiate the transcription of inflammatory genes. Recent studies have shown that the virulence protein YopJ activates the receptor-interacting protein kinases (RIPIs) and caspase-8 by inhibiting the activity of transforming growth factor kinase (TAK1), and activated caspase-8 can cleave GSDMD to induce pyroptosis [142, 143]. Interestingly, in 2013, Kang [144] found that caspase-8^−/−^ dendritic cells can produce more IL-1*β* than normal dendritic cells upon activation of the NLRP3 inflammasome induced by LPS, indicating that caspase-8 has a negative regulatory effect on LPS-induced NLRP3 inflammasome activation and IL-1*β* secretion. Further mechanistic studies have shown that the spontaneous activation of NLRP3 inflammasomes caused by LPS stimulation is dependent on RIPK1 and RIPK3, while knocking out RIPK3 in caspase-8^−/−^ dendritic cells can inhibit the release of IL-1*β* [154]. The same results were observed using RIPK3 kinase inhibitors. After knocking down the MLKL gene with small interfering RNA (siRNA), the same result, inhibited IL-1*β* secretion, is also apparent. All these studies have proven that caspase-8 negatively regulates the activation of the NLRP3 inflammasome through RIPK1-RIPK3-MLKL. Interestingly, using animal models, Andrew et al. [155] found that knocking out caspase-8 in mouse basal epidermal-forming cells can induce chronic skin inflammation and does not depend on the function of dermal macrophages, TNF, IL-1*β*, Toll-like receptor MyD88, TRIF, or other proteins. Another study found that in keratinocytes lacking caspase-8, interferon regulatory factor 3 (IRF3) and TANK-binding kinase 1 (TBK1) showed continuous phosphorylation and induced higher levels of IFN-*β* and IFN-inducing proteins. However, this inflammatory response was reversed when the level of IRF3 was deficient.

### 5.4. Contribution of Casapse-12 to Pyroptosis

As a member of the inflammatory caspase family, caspase-12 is expressed in a variety of tissues and plays an important regulatory role in the initiation and development of pyroptosis [147, 156]. In 2006, Saleh et al. [157] found that caspase-12 can play a negative regulatory role in the process of caspase-1 activation. Moreover, it was proven through mechanistic experiments that the activation of caspase-1 and the secretion of IL-1*β*/IL-18 are closely related to the enzyme activity state and protein size of caspase-12. Through further exploration, researchers found that caspase-12 and caspase-1 can structurally interact, thereby inhibiting the activity of caspase-1 [158]. In addition, a study by Walle et al. [159] revealed that neither cells nor mice lacking caspase-12 showed an increase in the activation ability of caspase-1 and in the secretion of IL-1*β*/IL-18. These two studies show conflicting results. Therefore, the contribution of caspase-12 to the regulatory mechanism of pyroptosis needs to be clarified with further studies.

## 6. The Roles of Gasdermin (GSDM) Family Proteins in Pyroptosis

### 6.1. Classification of GSDM Family Proteins

The GSDM protein family is a class of conserved proteins in vertebrates, including gasdermin A (GSDMA), gasdermin B (GSDMB), gasdermin C (GSDMC), gasdermin D (GSDMD), gasdermin E (GSDME, DFNA5), and Pejvakin (DFNB59). Among these family members, GSDMA, GSDMB, GSDMC, and GSDMD are homologous. Mice lack GSDMB [160, 161] but express three GSDMA homologs, GSDMA1, GSDMA2, and GSDMA3, and four GSDMC homologs, GSDMC1–4. Except for DFNB59, which contains only the N-terminal domain, all other GSDM proteins are composed of a C-terminal inhibitory domain (RD) with self-inhibition and protection functions and an N-terminal effector domain (PFD) that induces cytotoxicity. All GSDM family proteins can induce pyroptosis, and 45% of the genes in this family are homologous. However, only the GSDMD protein in the GSDM protein family involved in the induction of pyroptosis is relatively clear.

### 6.2. The GSDM Family Proteins Involved in Pyroptosis and Their Mechanisms

GSDMD is a protein of 480 amino acids with two domains at the N-terminus and C-terminus (the N-terminus is 242 amino acids long with an extended *β*-sheet core structure, the C-terminus is 199 amino acids long with *α*4-helix globular folds, and a 43-amino acid linker with a caspase cleavage site connects the two domains through a pocket-like structure) [162, 163]. Since 2015, the role of GSDMD in the process of pyroptosis has been gradually clarified. GSDMD is cleaved and activated as a substrate of caspase-1, 4, 5, or 11. Cleaved GSDMD forms an N-terminal effector domain (GSDMD-N) of 30 kDa and a C-terminal inhibitory domain (GSDMD-C) of 23 kDa, which releases the self-inhibition state. GSDMD-N can bind to membranes containing cardiolipin (CL) (in prokaryotic cells) and phosphatidylinositol (PI) (in prokaryotic cells) through Glu15 and Leu56 amino acid sites, undergoing oligomerization and resulting in the destruction of cell membrane integrity [12, 164]. Moreover, researchers using high-resolution atomic force microscopy analysis further revealed a series of dynamic actions of the GSDMD-N-terminal protein in the cell membrane, including membrane insertion, polymerization, and assembly into channels [11, 165]. In addition, GSDMD-N can act on mitochondria to promote the production and release of ROS, act on inflammasomes to activate downstream pathways to trigger the production and secretion of proinflammatory cytokines, and exert immune defense functions [119, 166]. Previous studies [167] have also shown that GSDMA3 has a high degree of structural similarity with GSDMD and unactivated GSDMA3 is in a self-inhibited state. However, disulfiram, a drug for treating alcohol addiction, was identified as an inhibitor of pore formation by GSDMD, rather than other members of the GSDM family. Moreover, disulfiram covalently modifies human/mouse Cys191/Cys192 in GSDMD to block pore formation at nanomolar concentration [168]. In addition, Karmakar and colleagues found that in contrast to macrophages activated by inflammasomes, biochemical and microscopy studies reveal that GSDMD-N in neutrophils predominantly associates with azurophilic granules and LC3^+^ autophagosomes [19].

The cell membrane damaged by the insertion of GSDMD-NT disrupts the normal permeability barrier of the plasma membrane and the normal separation of sodium and potassium [169, 170]. Under normal circumstances, the cytoplasm contains a higher concentration of potassium and a lower concentration of sodium but these proportions are opposite in the extracellular fluid and the concentrations differ at the two sides of the plasma membrane. Therefore, when the plasma membrane ruptures, the concentration gradient enables a large amount of potassium to move from the cytoplasm into the external fluid. In contrast, Na^+^ are absorbed into cells through the concentration gradient to the same degree as potassium efflux, causing a large amount of sodium ions to accumulate in the cell. A large volume of water from the extracellular fluid enters the cell instantaneously, resulting the cell to swell.

GSDME was recently identified to be an executioner protein for pyroptosis induced by chemotherapeutic drug [171, 172]. The N-terminal and C-terminal domains of the GSDME protein each contain a site that can be recognized and cleaved by caspase-3, which can induce pyroptosis upon activation [173]. The process is similar to that of GSDMD protein induction of pyroptosis, but the upstream molecules are obviously different from those activated in the GSDMD-dependent pathway. Under normal circumstances, a variety of clinical antitumour chemotherapeutics depend on the mitochondrial pathway to activate caspase-3 and induce apoptosis. However, GSDME can induce pyroptosis even in tissues with normal expression, which in turn leads to inflammatory damage, a damage that is widely recognized as an important reason for the side effects of chemotherapy drugs, to related tissues [174]. Yu et al. [175] found that lobaplatin-induced pyroptosis of colon cancer cells is dependent on GSDME and knocking out GSDME leads to the conversion of lobaplatin-induced pyroptosis to apoptosis but it does not affect the inhibitory effect of lobaplatin on cell growth in vitro or on tumor formation in vivo.

GSDMB can also be activated by caspase-3, 6, and 7 in a manner similar to GSDMD, and some researches have shown that GSDMB overexpression occurred in several kinds of cancers [176, 177]. In addition, Chen and colleagues suggested that overexpression of GSDMB promotes GSDMD cleavage, accompanied by increased LDH release, and further found that GSDMB promotes caspase-4 activity, which is required for the cleavage of GSDMD in noncanonical pyroptosis, by directly binding to the CARD domain of caspase-4 [178]. As for GSDMA, GSDMC, and DFNB59, the activation conditions and related mechanisms urgently need to be confirmed by research.

## 7. The Regulation of lncRNAs in Pyroptosis

**Figure 4:**
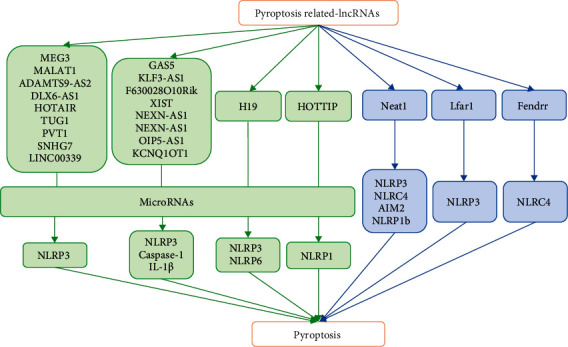
The ways of lncRNAs in regulating pyroptosis. lncRNAs are involved in the regulation of pyroptosis by indirectly acting on miRNAs and downstream targets or directly binding to procaspase-1 and thereby regulate the physiological process of pyroptosis-related diseases.

Long noncoding RNA (lncRNA) refers to a noncoding RNA with a transcript greater than 200 nt in length [179]. To date, hundreds of long noncoding RNAs have been discovered through transcriptomic and traditional microarray methods [180]. lncRNAs play important roles not only in the development and differentiation of innate immune cells but also in infection-related diseases. ln recent years, due to the rapid development and wide application of second-generation sequencing technology, the recognition level and mechanism research of lncRNA has advanced to a new level [181]. However, the understanding of the lncRNA regulatory mechanism is still in its infancy due to its complex regulatory mechanism. In addition, as an important natural immune response, pyroptosis not only plays an important role in the body's defense against pathogen invasion but also serves as bridges connecting adaptive immunity. Previous evidence has shown that lncRNAs are involved in gene regulation, X chromosome inactivation, genome imprinting, cell differentiation and development, and other biological processes [182–185] and can be widely expressed in monocytes, macrophages, dendritic cells, and neutrophils. In recent years, increasing evidence has shown that lncRNAs can participate in the regulation of pyroptosis, but few studies have focused on their specific regulatory mechanism. lncRNAs have recently been shown to play important roles by directly or indirectly acting on proteins related to the pyroptosis signalling pathway. Thus, lncRNAs participate in the pathological processes of cardiovascular disease, kidney disease, immune disease, and other related diseases (Figure 4). Zhang et al. [186] found that melatonin can attenuate ox-LDL-induced pyroptosis of human aortic endothelial cells by downregulating lncRNA MEG3, indicating that lncRNAs can promote pyroptosis. Research by Li et al. [187] showed that downregulation of the lncRNA MALAT1 can inhibit the activation of NLRP3, thereby attenuating human renal tubular epithelial cell pyroptosis. Studies have also shown that the lncRNA GAS5 can regulate the expression of pyroptosis-related molecules (such as ASC, caspase-1, IL-1*β*, and IL-18) in ovarian cells [188]. lncRNA KCNQ1OT1 can increase the level of caspase-1 by sponging miR-214 to regulate the occurrence and development of cataracts [189]. Another study has shown that low-dose chronic erucic acid can reduce the pyroptosis rate of macrophages in diabetic atherosclerotic rats by downregulating the lncRNA MALAT1 [190]. lncRNA Neat1 can promote the assembly of inflammasomes (NLRP3, NLRC4, AIM2, and NLRP1b) that rely on caspase-1 to mediate canonical pyroptosis and can also stabilize the interaction of mature caspase-1 heterotetramer intermediate subunits, thereby stabilizing and increasing the enzyme activity of caspase-1. The final result is that the lncRNA Neat1 promotes the activation of inflammasomes, the activation of caspase-1, and the maturation and secretion of proinflammatory cytokines, which accelerate the inflammatory response of mouse macrophages [191]. Overexpression of lncRNAKLF3-AS1 in exosomes can lead to reduction of the myocardial infarction (MI) area, decreased cell apoptosis and pyroptosis, and attenuated MI progression and can also indirectly regulate the pyroptosis of cardiomyocytes in vivo and in vitro by sponging miR-138-5p [192]. Recent studies have shown that inhibiting lncRNA-H19 can effectively inhibit pyroptosis and this promotion of pyroptosis is achieved by a competing endogenous RNA network (ceRNET) formed by lncRNA-H19 through the sponge miR-21 to promote PDCD4 expression and inhibiting H19 will be a reliable treatment [193]. Moreover, there are reports showing that the in vitro knockdown of lnc-Lfar1 significantly suppressed LPS- and IFN-*γ*-induced proinflammatory activation of macrophages and inhibited LPS/ATP- and LPS/nigericin-induced NLRP3 inflammasome-mediated pyroptosis. Mechanistically, lnc-Lfar1 regulated LPS- and IFN-*γ*-induced proinflammatory activation of macrophages through the NF-*κ*B pathway [194]. lncRNA F630028O10Rik functioned as a ceRNA for the miR-1231-5p/Col1a1 axis and enhanced microglial pyroptosis after the secondary phase of spinal cord injury (SCI) by activating the PI3K/AKT pathway. Furthermore, STAT1 was the upstream transcriptional factor of IncRNA-F630028O10Rik and was induced by the damage-responsive TLR4/MyD88 signal [195]. lncRNA ADAMTS9-AS2 acted as a tumor suppressor in gastric cancer (GC) cells by activating NLRP3-mediated pyroptotic cell death through sponging miR-223-3p [196]. Knockdown of lncRNA-XIST inhibited NSCLC progression by triggering miR-335/SOD2/ROS signal pathway-mediated pyroptotic cell death [197]. Another recent study proposed that knockdown of lncRNA DLX6-AS1 inhibits HK-2 cell pyroptosis via regulating the miR-223-3p/NLRP3 pathway in lipopolysaccharide-induced acute kidney injury [198]. In addition, it has also been reported that NLRC4 and inflammatory cytokines associated with pyroptosis were decreased in the high-glucose-treated hypoxia/reoxygenation- (H/R-) induced microglia after lncRNA-Fendrr knockdown [199]. Moreover, lncRNA HOTAIR silencing significantly inhibits neuronal damage through repressing NLRP3-mediated pyroptosis activation via regulation of the miR-326/ELAVL1 axis in Parkinson's disease (PD), which may contribute to a better understanding of PD pathogenesis and provide new treatment strategies for this disease. There are studies showing that overexpression of lncRNA TUG1 alleviates NLRP3 inflammasome-mediated cardiomyocyte pyroptosis through targeting the miR-186-5p/XIAP axis in coronary microembolization- (CEM-) induced myocardial damage [200]. lncRNA PVT1 modulates NLRP3-mediated pyroptosis in septic acute kidney injury by targeting miR-20a-5p [201]. Long noncoding RNA SNHG7 inhibits NLRP3-dependent pyroptosis by targeting the miR-34a/SIRT1 axis in liver cancer [202]. Long noncoding RNA LINC00339 promotes renal tubular epithelial pyroptosis by regulating the miR-22-3p/NLRP3 axis in calcium oxalate-induced kidney stone [203]. lncRNA HOTTIP inhibits cell pyroptosis by targeting the miR-148a-3p/AKT2 axis in ovarian cancer [204]. lncRNA OIP5-AS1 knockdown or miR-223 overexpression can alleviate LPS-induced ALI/ARDS by interfering with miR-223/NLRP3-mediated pyroptosis [205]. In addition, atorvastatin can drive pyroptosis and its inhibitory effects on pyroptosis are markedly offset by knockdown of lncRNA NEXN-AS1 or interference of NEXN [206]. In recent years, an increasing number of studies have proven that additional lncRNAs are involved in the regulation of pyroptosis-induced diseases. However, since the research of lncRNAs in immunity has recently begun, there are still many difficulties and problems that need to be solved urgently in the study of lncRNA's involvement in pyroptosis. For example, whether lncRNAs can be targeted, similar to miRNA, to mediate pyroptosis in many pathological processes by inhibiting, silencing, or promoting gene expression and whether lncRNAs can directly participate in the regulation of a certain pathway of pyroptosis through specific mechanisms remain to be further studied.

## 8. Negative Regulation of NLRP3

A moderate inflammatory response is conducive to the elimination of pathogenic microorganisms in the body, while excessive or abnormal inflammatory responses can cause autoimmune diseases. Therefore, while efficiently removing pathogenic microorganisms, inflammation must be controlled and stopped before it inflicts damage on tissues. Autophagy, as a response of cells to starvation, can transfer damaged organelles and long-lived proteins phagocytosed by autophagosomes from the cytoplasm to lysosomes for degradation. A large number of recent studies have revealed multiple roles of autophagy in regulating cell death, differentiation, antigen presentation, and antimicrobial responses [207]. Autophagy was proven to be an inhibitor of the NLRP3 inflammasome in Atg16 L1^−/−^-deficient mice. The absence of Atg16 L1 prevented autophagy but enhanced the activation of inflammasomes induced through TLR4 signal transduction [208]. In addition, type I interferon can also inhibit the production of IL-1*β* through two different mechanisms. On the one hand, the amount of caspase-1-dependent IL-1*β* secreted through the mediation of the transcription factor STAT1 is reduced by inhibiting the activities of inflammasomes NLRP1 and NLRP3; on the other hand, type I interferon produces IL-10 mediated by STAT1 to reduce the abundance of IL-1*β*; in turn, IL-10 can also inhibit the synthesis of pro-IL-1*β* and pro-IL-1*α* through the STAT3 signalling pathway [209]. Furthermore, the adaptive immune system can also inhibit the innate immune system. Effector and memory CD4^+^ T cells can inhibit inflammasome-mediated caspase-1 activation and IL-1*β* secretion from macrophages and dendritic cells (DCs). However, this inhibition requires cell-to-cell contact and is mediated by TNF superfamily ligands such as CD40 L, OX40 L, and RANKL [210] and the specific interaction mechanism between this ligand and cell receptors is still unclear. In addition, the TRIM protein family has also been proven increasingly by evidences that it can regulate the activation of inflammasomes. TRIM30 is a TRIM protein containing a RING domain, a negative regulator of TLR signalling, and can specifically inhibit the activation of NLRP3 endosomes [211]. Moreover, there is also a microRNA that can regulate the activation of inflammasomes, specifically by targeting the NLRP3 3′-UTR to prevent the accumulation of NLRP3 protein, and miR-223 can be used as a modulator to inhibit NLRP3 inflammasome activation [212]. Studies have also shown that pyrin-only proteins (POPs) and CARD-only proteins (COPs) can act as regulators of human inflammasome activation. POP interferes with the PYD-PYD interaction between NLRP and the adaptor protein ASC to reduce inflammation [213–215]. In contrast to POP, COP has a function similar to that of the CARD in caspase-1. COP can interact with CARD to act as a decoy inhibitor of caspase-1 to isolate it from the upstream-activated adaptor protein and exert an inhibitory effect. Nitric oxide (NO), as a small molecule synthesized by tissue cells, can participate in a variety of physiological and pathological processes [216]. Recent research has shown that NO can inhibit the formation of NLRP3 inflammasomes, thereby attenuating ASC recruitment, caspase-1 activation, and IL-1*β* secretion from mouse and human bone marrow cells, while a lack of iNOS enhances the activation of NLRP3 through mitochondrial dysfunction. The activation of NLRP3 inflammasomes by microbes can be inhibited in a variety of ways to promote infection. For example, Orf63 encoded by KSHV, a virus homologue of NLRP1, can block NLRP1-dependent innate immune response-activation of caspase-1 and the processing of IL-18 and IL-1*β* [72]. Other studies have shown that measles virus (MV) antagonizes the host's innate immune response through nonstructural protein V. The V protein of MV can interact with NLRP3 through its C-terminal domain, thereby inhibiting the secretion of IL-1*β* mediated by the NLRP3 inflammasome [217]. Furthermore, our research group is also conducting research studies to determine whether *Aeromonas veronii* activates the host's innate immunity. Since this is a pathogen of aquatic organisms, previous research has mainly focused on fish and have shown that the bacteria can cause EPCs (epithelioma papulosum cyprinid) to swell and round. Moreover, similar related results have been published in relevant journals. To further explore its specific induction mechanism, our research group is conducting related research on pyroptosis. Since *A. veronii* is similar to *E. coli* and both are Gram-negative bacteria, many studies on the ability of *E. coli* to induce pyroptosis have been reported and mammals including humans can also be infected with *A. veronii* causing sepsis similar to aquatic organisms. Therefore, we preliminarily speculate that *A. veronii* may activate the host's innate immune response and subsequent pyroptosis but its specific mechanism needs to be further explored.

## 9. Concluding Remarks and Perspectives

As a recently proposed mode of programmed cell death that is distinct from apoptosis, pyroptosis plays central roles in pathogen clearance and host defense during the innate immunity. Host cells are frequently invaded by intracellular pathogens that are harboured in the pathogen-containing vacuoles or in the cytoplasm to replicate. Cell lytic death can directly eliminate these intracellular replication niches formed by pathogens, and meanwhile, the released infectious agents are exposed to the extracellular immune defense to be captured and killed by other immune cells and cytoplasm contents released by pyroptosis initiate the inflammatory cascade. Eventually, the initiation of local inflammation recruits and activates a large number of immune cells to accelerate the elimination of pathogens from the host.

Pyroptosis is a key component of the innate immune and plays essential roles in eliminating pathogenic infections and endogenous danger signals. As a form of cell death accompanied by inflammation, pyroptosis is no less important than that of apoptosis, especially in certain pathological processes, its actual effect may far outweigh apoptosis. The successful identification of gasdermin D, the substrate molecule of these inflammatory caspases and a pore-forming protein, at the downstream of the caspase-1-dependent and independent pathways to activate pyroptosis has opened up many new avenues for in-depth analysis of the function of pyroptosis after the occurrence of infectious diseases. However, research on the susceptibility of other gasdermin proteins and caspase proteins to infectious agents in mice is still lacking. The comparison of the responses to a series of infectious agents in mice lacking gasdermin D, caspase-1, and/or caspase-11 alone and in combination can clearly reveal the necessary contributions of these components in the defense of the host against infectious microorganisms. Moreover, long noncoding RNAs participate in the occurrence and development of diseases by regulating the proinflammatory or anti-inflammatory functions of targeted genes during this inflammatory response. Therefore, the further in-depth study and function clarification of lncRNAs in the process of pyroptosis will facilitate to understand the detailed molecular mechanism of related diseases and are expected to provide new targets for the diagnosis of inflammatory and new therapeutic targets for the infectious diseases.

## Figures and Tables

**(a):** 

PRRs	Localization	PAMP recognized	Key adaptors	Effector response
*TLRs*				
TLR1	Cell surface	Triacylated lipopeptides	MyD88	IL-6, TNF-*α*
TLR2	Cell surface	Di/triacylated lipopeptides	MyD88, TIRAP	IL-6, TNF-*α*, MCP-1, RANETS
TLR3	Endosomes	dsRNA	TRIF	IFN-*β*
TLR4	Cell surface	LPS	MyD88, TRIF, TIRAP, TRAM	IL-6, TNF-*α*, IFN-*β*, IP-10
TLR5	Cell surface	Flagellin	MyD88	TNF-*α*
TLR6	Cell surface	Diacylated lipopeptides	MyD88, TIRAP	TNF-*α*, IL-6, IL-8, MCP-1, RANTES
TLR7	Endosomes	ssRNA	MyD88	IFN-*α*
TLR8	Endosomes	ssRNA	MyD88	IFN-*α*
TLR9	Endosomes	CpG DNA	MyD88	IFN-*α*
TLR11	Endosomes	Profilin, flagellin	MyD88	IL-12, TNF-*α*
TLR12	Endosomes	Profilin	MyD88	IL-12p40, IFN-*α*
TLR13	Endosomes	23s rRNA	MyD88	IL-6, IL-12p40

**(b):** 

Subfamily	Gene	Localization	PAMP recognized	Activator	Effector response
NLRA	CIITA	Cytoplasm		IFN-*γ*	
NLRB	NAIP	Cytoplasm	Triacylated lipopeptides	Flagellin	IL-6, TNF-*α*
NLRC	NOD1	Cytoplasm	Di/triacylated lipopeptides	DAP	IL-6, TNF-*α*, MCP-1, RANETS
	NOD2	Cytoplasm	dsRNA	MDP	IFN-*β*
	NLRC3	Cytoplasm	LPS	Unknown	IL-6, TNF-*α*, IFN-*β*, IP-10
	NLRC4	Cytoplasm	Flagellin	Rod protein, flagellin	TNF-*α*
	NLRC5	Cytoplasm	Diacylated lipopeptides	IFN-*γ*, IFN-*β*	TNF-*α*, IL-6, IL-8, MCP-1, RANTES
	NLRX1	Cytoplasm	ssRNA	Unknown	IFN-*α*
NLRP	NLRP1	Cytoplasm	ssRNA	Lethal Toxin, MDP	IFN-*α*
	NLRP2	Cytoplasm	CpG DNA	Unknown	IFN-*α*
	NLRP3	Cytoplasm	Profilin, flagellin	ATP, Alum, Asbestos, Silica, ROS	IL-12, TNF-*α*
	NLRP4	Cytoplasm	Profilin	Unknown	IL-12p40, IFN-*α*
	NLRP5	Cytoplasm	23s rRNA	Unknown	IL-6, IL-12p40
	NLRP6	Cytoplasm	Colitis, colorectal tumorigenesis	Unknown	NF-*κ*B, ERK, IFN-*α*,
	NLRP7	Cytoplasm	Unknown	Lipopeptides	IL-18, IL-1*β*
	NLRP8	Cytoplasm	Unknown	Unknown	Unknown
	NLRP9	Cytoplasm	Unknown	Unknown	Unknown
	NLRP10	Cytoplasm	Unknown	Unknown	NF-*κ*B, IL-1*β*
	NLRP11	Cytoplasm	Unknown	Unknown	Unknown
	NLRP12	Cytoplasm	Unknown	Yersinia	NF-*κ*B
	NLRP13	Cytoplasm	Unknown	Unknown	Unknown
	NLRP14	Cytoplasm	Unknown	Spermatogenesis	Unknown

**Table 2:** Classification and expression pattern of the gasdermin family.

Mouse gene	Human gene	Alternate names	Expression	Activated by
Gasdma1, Gasdma2, Gasdma3	GSDMA	Gasdermin (GSDM), gasdermin1 (GSDM1)	Skin, tongue, esophagus, stomach, mammary glands, and umbilical cord	Unknown
Absent	GSDMB	PRO2521, gasdermin like (GSDML)	Lymphocytes, esophagus, stomach, liver, and colon	Unknown
Gsdmc1, Gsdmc2, Gsdmc3, Gsdmc4	GSDMC	MLZE	Esophagus, stomach, trachea, spleen, intestines, bladder, and skin	Unknown
Gsdmd	GSDMD	GSDMDC1, DFNA5L	Immune cells, stomach, esophagus, and intestines	Caspase-1/11/4/5
Dfna5	DFNA5	Gasdermin E (GSDME)	Placenta, brain, heart, kidney, cochlea, intestines, and IgE-primed mast cells	Caspase-3
Dfnb59	DFNB59	Pejvakin, PJVK	Inner ear hair cells, auditory system, broadly expressed in other tissues	Unknown
